# Easy-to-Use MOX-Based VOC Sensors for Efficient Indoor Air Quality Monitoring

**DOI:** 10.3390/s24082501

**Published:** 2024-04-13

**Authors:** Radu Nicolae Pietraru, Maximilian Nicolae, Ștefan Mocanu, Daniel-Marian Merezeanu

**Affiliations:** Faculty of Automatic Control and Computers, National University of Science and Technology Politehnica Bucharest, 060042 București, Romania; max.nicolae@upb.ro (M.N.); stefan.mocanu@upb.ro (Ș.M.); daniel.merezeanu@upb.ro (D.-M.M.)

**Keywords:** VOC sensors, IAQ monitoring, MOX-based sensors, low-cost sensors, sensor testing

## Abstract

This research paper presents a case study on the application of Metal Oxide Semiconductor (MOX)-based VOC/TVOC sensors for indoor air quality (IAQ) monitoring. This study focuses on the ease of use and the practical benefits of these sensors, drawing insights from measurements conducted in a university laboratory setting. The investigation showcases the straightforward integration of MOX-based sensors into existing IAQ monitoring systems, highlighting their user-friendly features and the ability to provide precise and real-time information on volatile organic compound concentrations. Emphasizing ease of installation, minimal maintenance, and immediate data accessibility, this paper demonstrates the practicality of incorporating MOX-based sensors for efficient IAQ management. The findings contribute to the broader understanding of MOX sensor capabilities, providing valuable insights for those seeking straightforward and effective solutions for indoor air quality monitoring. This case study outlines the feasibility and benefits of utilizing MOX-based sensors in various environments, offering a promising avenue for the widespread adoption of user-friendly technologies in IAQ management.

## 1. Introduction

Air quality is one of the most important factors influencing people’s health. Air quality monitoring is a tool that prevents decrease in quality of life and people’s health. There are several differences between outdoor and indoor air quality monitoring caused by the types of polluting agents and how they affect people’s health. Outdoor air quality is a highly investigated problem because it affects both people’s health and especially the health of the planet. Outdoor air pollution is closely related to climate change and damage to various ecosystems, which directly affects people’s health and well-being. However, people spend most of their time indoors; according to [1,2], between 80% and 90% of the time is spent indoors by the majority of people. For this reason, indoor environmental pollution represents a strong threat to human health and less of a direct threat to the outdoor environment. Moreover, air pollution in personal homes is an individual problem more than a society problem. Indoor air quality monitoring systems are not mandatory anywhere in the world for private homes but only in certain areas, for office spaces, shops, or other public or industrial indoor spaces. Being at the discretion of each individual, the effective implementation of air quality monitoring systems in the personal home can be affected by factors regarding the cost [3,4], as well as by the understanding of the indoor pollution phenomenon. The comparative analysis of VOC sensors based on metal-oxide technology (MOX-based) presented in this paper aims to contribute to the implementation of indoor air quality monitoring systems at ultra-low costs that are easy to understand and used by different users. The choice of MOX sensors is easy to make if we consider various studies that reveal their popularity. In particular, ref. [5] claims that MOX sensors are used in over 35% of applications based on low-cost sensors, while other studies [6,7] point out their characteristics and advantages more briefly.

Indoor air quality is defined by several parameters, including the concentration in the air of various harmful gases such as carbon monoxide and dioxide (CO and CO_2_), ozone (O_3_), nitrogen oxides (NO_x_), volatile organic compounds (VOCs), and the concentration of matter particles with various diameters (PM_2.5_, PM_10_). Most commercial indoor air quality monitoring systems measure only one or a few of these parameters for reasons related to cost and complexity of implementation. For example, air filtration systems only measure the concentration of matter particles, and automatic ventilation systems only measure the concentration of gaseous pollutants (CO_2_ or VOC). This way, the cost and complexity of the systems are lower and become more accessible to a larger number of users.

Even if the concentration of volatile organic compounds (VOCs) is a parameter that can be monitored both indoors and outdoors [8], the impact on air quality is primarily monitored indoors. There is even an equivalence (explored in the analysis carried out in this paper) between the concentration of volatile organic compounds and the concentration of CO_2_ indoors [9,10]. The name used for CO_2_ proportional to the VOC concentration is eCO_2_ (equivalent CO_2_).

Another extremely important reason why the concentration of VOCs in indoor air is an essential element is the strong effect on people’s health being closely related to Sick Building Syndrome (SBS)—a series of negative clinical manifestations that have no other medical explanations than those generated from poor indoor air quality. Precisely due to the effects on people’s health, the role of VOC concentration in indoor air quality has been the subject of analysis and regulation at government level [11,12,13] during the last 20 years, and safety limits have already been defined for newly constructed buildings [14].

This paper tries to explore the currently available VOC sensor solutions that allow the design and construction of easy-to-use and low-cost monitoring systems, but which also allow efficient indoor air quality monitoring. In this paper, a comparative analysis of ten digital MOX-based low-cost VOC sensors is carried out to see what options exist in the current design of low-complexity and low-cost monitoring systems. The comparative analysis carried out in this paper does not aim to verify the performance of the tested sensors, but only their ease of use. Even if the tests carried out inherently aim at the main functionality of the sensors (TVOC concentration measurement), the scope of the research in this paper is to see how the sensors behave within a monitoring prototype and if a similar behavior can be reached regardless of the used sensors. An easy usage requires a similar set of features specific to the field of use, IAQ monitoring. Section 2 of the paper presents some relevant related works within the field of indoor air quality monitoring systems as well as other studies carried out to improve the design of air quality monitoring systems. Section 3 presents the ten sensors analyzed in the paper as well as the test methodology. The data obtained after testing and the comparative analysis of the ten sensors are presented and discussed in Section 4 of the paper. Section 5 exhibits the authors’ conclusions drawn from the research carried out and presented in the current paper.

## 2. Related Works

Sensors, from the moment they were created, evolved and offered the possibility of monitoring various physical, chemical, meteorological, or other parameters. In time, not only have they allowed the possibility of collecting data from processes with the aim of local automatic control, but they have also allowed humans to informally/virtually be present in places that are not safe for life or physical integrity. With the aid of various communication protocols, architectures like WSN (Wireless Sensor Network) and IoT (Internet of Things) have emerged and evolved, and now they are strongly related and omnipresent when it comes to fields like remote real-time data acquisition [15,16,17], storage [16], control [18], big data [19,20], data logging [20,21], data mining/machine learning [20,21], cloud/edge/fog computing [21,22,23,24].

The COVID-19 pandemic represented a significant landmark in terms of air quality monitoring in various indoor spaces. From the point of view of the physical educational environment, the COVID-19 pandemic emphasized the need for good management of the resources represented by classrooms and laboratories. In order to ensure a safe working environment, social distancing was an easy-to-manage measure, but it could not guarantee the quality of some internal environment parameters. For this reason, using air quality monitoring sensors has resulted in a simple, cheap, and safe way to collect indoor environment data and use it for making fast and effective decisions (starting HVAC systems, room evacuation, alerts, and so on) [25,26]. Another direction consists of using indoor data to identify patterns and create scenarios that allow preemptive actions [27,28,29,30]

When it comes to indoor air quality, VOC concentration is a representative parameter that can be used alone in cases of low-cost and low-complexity monitoring systems. Authors of [31] focused both on analyzing VOCs and TVOCs (Total Volatile Organic Compounds) starting from the observation that there is no standardized approach worldwide. However, their conclusions and recommendations indicate that individual VOCs should, in general, be considered rather than TVOCs. By aggregating several VOCs into one TVOC, one may be exposed to the risk of reducing the actual impact of a specific VOC that has a low numerical value but a very high impact over health. On the other hand, authors of [32] mainly focused on TVOC in their study and consider that TVOC is a relevant parameter for building benchmarking. They also showed how pollution could be significantly reduced in the most polluted space they analyzed.

The differences between the sensors used in current IoT applications dedicated to similar purposes are related, without limitation, to construction, cost, precision, communication protocols, interface, etc. While the internal architecture of a sensor is usually of very low importance for the user, the others may make a significant difference. In paper [33], the authors conducted an analysis solely based on a comprehensive bibliography. In the end, they managed to offer an interesting characterization of various VOC sensors based on their construction, some technical features, strengths, and weaknesses. However, the authors did not investigate aspects related to communication protocols, interface, and ease of use. A similar approach was used by authors of [34], but they went a little further by indicating potential applications for the different types of analyzed sensors and, also, the manufacturers of the sensors. Just like in the previously referred study, the authors did not go into technical details related to actually using the sensors inside some applications.

Regarding the sensors nowadays and their use, one of the main challenges for a developer is to find a good balance between cost and precision. Is it really mandatory to spend a lot of money buying a very expensive sensor with a very high precision, or is it enough to have a decent, low-cost sensor, allegedly with a smaller precision? Papers [35] and [36] go deep into this problem and try to offer some useful insights. Even though the authors of the mentioned studies analyze different VOC sensors, the conclusions seem to support the same ideas: 1. Low-cost sensors should definitely be considered for use inside non-critical applications; 2. Calibration is very important and can lead to very good results; and 3. Low-cost sensors (LCSs) should be used with caution, since they are often sold by their manufacturer without guarantees related to use, reliability, or performance.

The final aspects that must be evaluated prior to integrating sensors in various applications are related to what and how sensors send data to the core of the architecture which, in most cases, is represented by a microcontroller. While microcontrollers have the ability to interact with analogue devices, most developers prefer digital ones. As authors present in paper [37], there are a few advantages offered by analogue sensors (some of them being the measurement range and continuous reading), but there are more disadvantages when it comes to the need of using an external analogue-to-digital converter, overall costs, and more difficult integration. The conclusion of the mentioned paper is that, in most cases, digital sensors are preferred. The same conclusion is reached by the authors of [38], who also offer reliable information on how to overcome the minor problems generated by the digital sensors and gain the most from their advantages. From this point forward, the last important characteristic of a sensor (from the integration point of view) is to be considered, that is, interface. In paper [39], the authors make a thorough analysis of the most relevant communication protocols used today in embedded applications. From the perspective of this study, the conclusions related to I2C protocol are of high value, since this protocol offers a good compromise between communication speed, architecture, and usability.

## 3. Materials and Methods

### 3.1. Sensors

In recent years, there has been a notable trend in the field of indoor air quality (IAQ) monitoring towards transitioning from providing absolute values to presenting results in the form of an air quality index (AQI). This shift reflects a broader effort to enhance accessibility and comprehension of IAQ data among users from diverse backgrounds. By condensing complex measurements into a single, easily interpretable value, the adoption of an air quality index simplifies data presentation and facilitates standardized communication of air quality information to the public. However, this transition also introduces challenges, particularly regarding the loss of granularity and the reliance on sensor calibration for accuracy. In this context, it becomes imperative to explore both the advantages and disadvantages of this evolving approach to ensure the effective utilization of IAQ monitoring technologies. Besides this, manufacturers of TVOC sensors often incorporate temperature (Temp), relative humidity (RH), pressure, and particulate matter (PM) sensors into their products to enhance accuracy and reliability. By compensating for environmental variations, such as changes in temperature, humidity, and pressure, these integrated sensors ensure more precise measurements of VOC concentrations (as manufacturers claim). Alongside these sensors, TVOC sensors frequently include mechanisms for evaluating equivalent carbon dioxide (eCO_2_), which represents the total concentration of carbon-containing gases in the air as if they were carbon dioxide. This comprehensive approach not only improves the overall performance of TVOC sensors but also expands their applicability across a range of dynamic environments, including indoor air quality monitoring in buildings and outdoor environmental monitoring. Additionally, the integration of multiple sensors into a single device simplifies installation and operation for users, making TVOC sensors with integrated environmental sensors a convenient and effective solution for comprehensive environmental monitoring needs.

The choice of sensors considered criteria such as popularity, market availability, price under $30, compatibility with the test system (electrical operating parameters, hardware and software requirements for using the sensor), and previous experience of the research team. All selection criteria supported the idea of ease of use in building IAQ monitoring systems. The ten selected sensors were considered to be a representative collection for the scope of the research. All ten sensors met the selection requirements and had a high availability for purchase at the beginning of the tests (June 2023). The first step towards easy use is easy purchase.

Table 1 lists the sensors assessed in our evaluation, along with their designated target physical parameters, initial official release dates, and the applications recommended by their respective manufacturers.

Before presenting the table containing synthetic information about the sensors, it is pertinent to provide some comments. It is notable that most of the analyzed sensors lack explicit specification of their dynamic range, expressed as measurement range. Only three of them additionally offer a specified range within which they were tested by the producer. As previously mentioned, our objective is not to compare sensors for recommendation purposes, but rather to illustrate whether, in general, these types of Metal Oxide Semiconductor (MOX) sensors are suitable for IAQ applications and the level of integration effort required in terms of ease of use.

As is well known, the measurement principle of MOX sensors involves heating layers of the Metal Oxide Semiconductor, resulting in changes in conductivity when exposed to gases. These changes are then adapted and captured by analog-to-digital converters (ADCs). Most sensors provide access to raw data from the ADC, leading some producers to express resolution as the resolution of the ADC. However, in cases where specified, the ADC output may be proportional to the logarithm of the resistance of the sensing material. Consequently, the resolution of VOC output may differ from the resolution of the ADC. Moreover, numerous complex factors interfere with the computation of VOC output, making characteristics such as sensitivity challenging to evaluate. Producers utilize various methodologies, often following standards such as ISO 16000-29 [50], but they struggle to precisely specify sensitivity, particularly in specific situations (e.g., increasing ethanol concentration from 5 to 10 ppm [49,51]). It is noteworthy that only one producer among the analyzed sensors provides a clear distinction between certain characteristics, such as resolution vs. sensitivity, response time vs. sampling time, and measurement range vs. specified range. Regarding accuracy, manufacturers typically limit their evaluation to device-to-device or sensor-to-sensor (S2S) accuracy due to inherent variability between individual sensors of the same model. This variability can stem from manufacturing disparities, environmental factors, calibration nuances, and sensor aging. By providing accuracy as S2S variability, producers acknowledge the inherent variability in sensor performance and provide users with a more realistic assessment of reliability and consistency in real-world applications. In the context outlined above, we introduce Table 2, which systematically compiles the key parameters that most accurately describe the sensors utilized in our study. It is important to note that manufacturers often denote parameters in a less formal manner, leaving room for potential confusion. As a result, certain cells in the table intentionally remain unfilled, either because we could not ascertain the specific value or because we harbored doubts regarding whether the parameter specified by the manufacturer aligns precisely with its formal definition. Notably, one parameter where ambiguity persists is the response time; in some instances, it remains uncertain whether the provided value pertains to the sensor’s sensitivity or its sampling time.

### 3.2. The Test Environment

**Figure 1:**
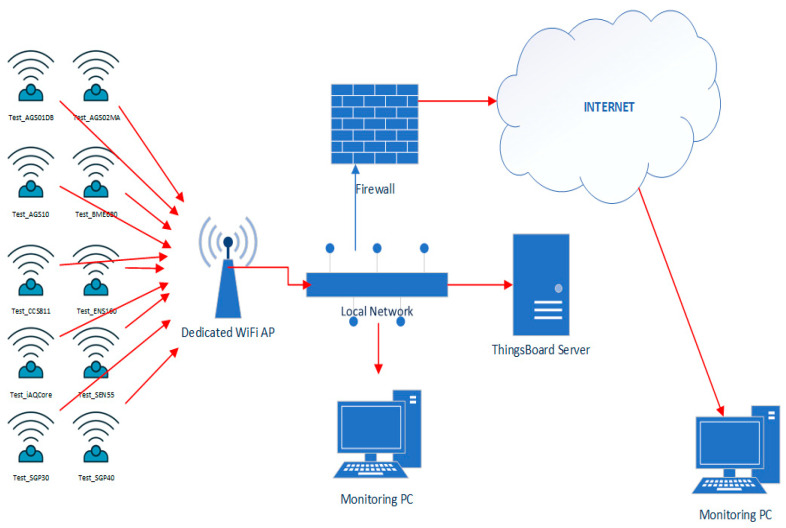
Test system architecture.

**Figure 2:**
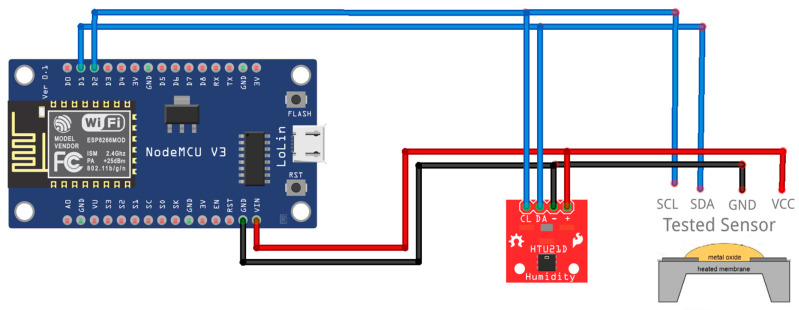
Interconnection diagram between the components of the test devices.

All the ten sensors tested are digital sensors with I2C output, while some have the alternative of higher speed interfacing through the SPI bus. To carry out the testing, a generic electronic test assembly was made consisting of a NodeMCU development board [61], equipped with a SOC WiFi Espressif ESP8266 microprocessor [62], an HTU21D temperature and humidity sensor, and the tested sensor. The role of the NodeMCU development board is to read the measured values from the two sensors and send them via WiFi to the ThingsBoard server (Figure 1) for recording. The HTU21D temperature and humidity sensor has the role of providing temperature and humidity to VOC sensors that require these values for an accurate measurement, for electronic compensation of environmental conditions. The HTU21D sensor was also used in the assembly for sensors that do not require this compensation. The HTU21D sensor was not used in the test schemes of the BME680 and SEN55 sensors, because these two sensors contain their own integrated internal temperature and humidity sensor. The HTU21D sensor is a digital sensor and shares the I2C bus with the tested sensor to communicate with the NodeMCU development board (Figure 2). All ten test systems were powered at a voltage of 5 V coming from a specialized power supply connected to the electricity network and able to provide a total power of 100 W.

The HTU21D sensor and the tested sensors were powered at 3.3 V, except for the SEN55 sensor which requires 5 V. The supply voltage of the tested sensors is the supply voltage of the modules containing the sensors; in some cases, it is not the actual supply voltage of the sensors. The voltage of 3.3 V was provided through the voltage regulator integrated into the NodeMCU board. Besides the two specific connections of the I2C bus (SDA and SCL lines), the following sensors needed specific connections: the SEN55 sensor had the SEL line connected to GND for the selection of the I2C type connection, the BME680 sensor had the SDO pin connected to GND for establishing of the I2C address, and the CCS811 sensor had the WAKE pin pulled to the ground to activate the sensor.

**Figure 3:**
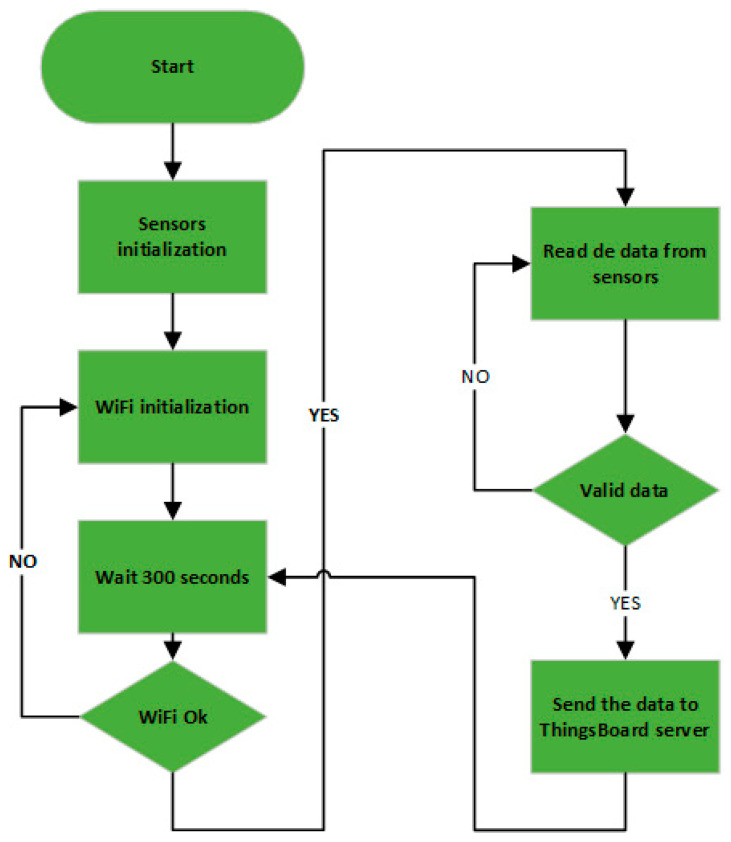
Execution diagram for the test device program.

The test systems were designed to go through an initialization phase (sensors and WiFi network) and then to work in an infinite loop of reading values from the sensors and sending the read values to the ThingsBoard server for recording (Figure 3). Reading and sending data was carried out at an interval of 5 min (300 s).

The programs for the monitoring devices were written using Arduino IDE 1.8.19 with ESP8266 Community extension 3.0.2 installed and the following libraries: Sensirion I2C SEN5x 0.3.0, BME68x Sensor library 1.1.40407, BSEC Software Library 1.8.1492, ArduinoJson 6.19.4, PubSubClient 2.8.0, Seed_Arduino3_0mbed_Arduino3.0.1, TBPubSubClient 2.9.1, and ThingsBoard 0.9.5.

**Figure 4:**
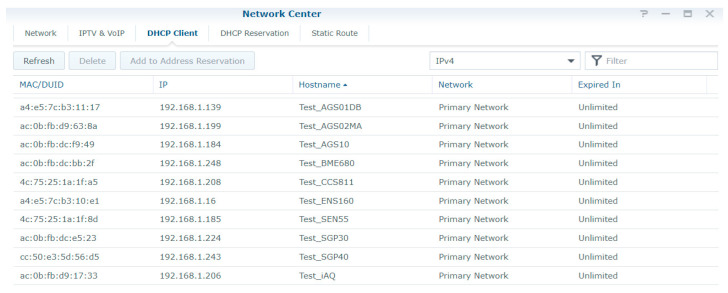
DHCP configuration for test WiFi devices.

**Figure 5:**
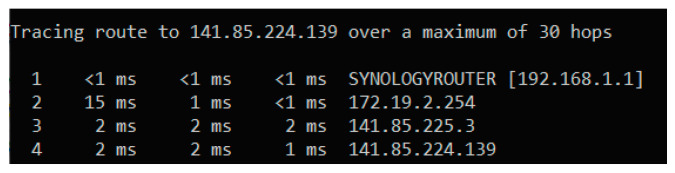
Testing the connection between the WiFi network of the test devices and the IP address of the ThingsBoard server.

To ensure a stable and secure WiFi connection for the test systems, a dedicated WiFi router was used only for the test systems. The router was configured to provide a WiFi network 802.11 b/g/n 2.4 GHz with WPA/WPA2 security in accordance with the communication capabilities of the NodeMCU development board. The test systems received the IP address with unlimited validity via DHCP in order not to cause unnecessary re-initialization of the WiFi connection (Figure 4). The ThingsBoard server was installed on a virtual server in the institution’s network (the data center of the National University of Science and Technology Politehnica Bucharest) to have minimum latency in data transmission and to avoid loss of measured data. The network tests performed showed a number of 4 hops in the institution’s local network and a maximum latency of 2 ms (Figure 5).

ThingsBoard is an open-source IoT platform [63] intended for the management of IoT devices, the recording and processing of data from IoT devices, and the creation of data visualization panels, and it has very good stability and scalability. In the case of the testing carried out in this paper, the ThingsBoard platform successfully fulfilled the function of recording the data sent via the MQTT protocol by the test devices and processing and analyzing the data for the tests performed and presented in Section 4. The ThingsBoard server was installed in the university datacenter on a virtual machine that had 4 processing cores at 2.2 GHz and 4 GB of RAM running CentOS 8. ThingsBoard version 3.6.2 Community Edition was used with a Postgresql 12 database.

**Figure 6:**
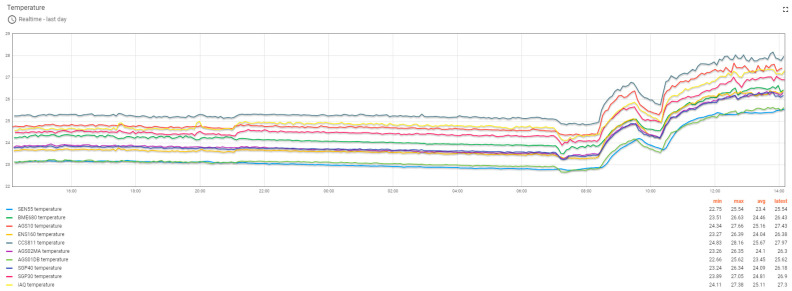
The temperature parameter values recorded on the last day for the ten test devices.

**Figure 7:**
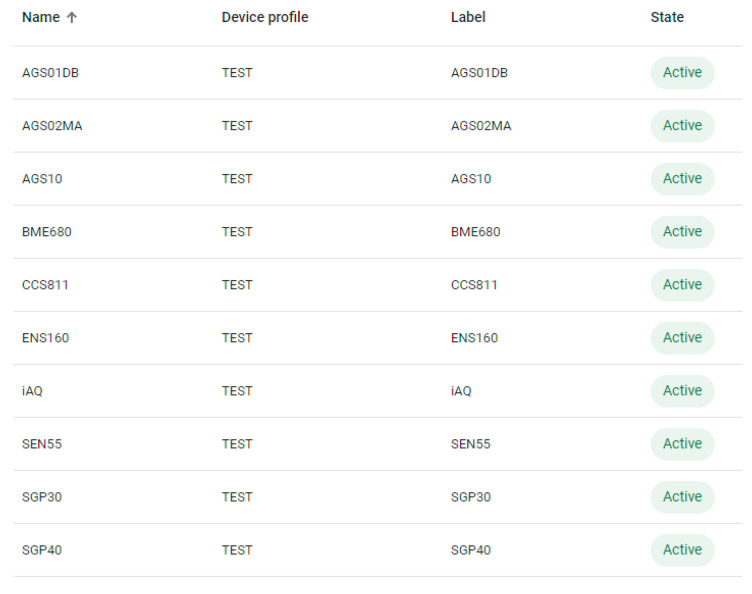
MQTT connection status between each test system and the MQTT server.

**Figure 8:**
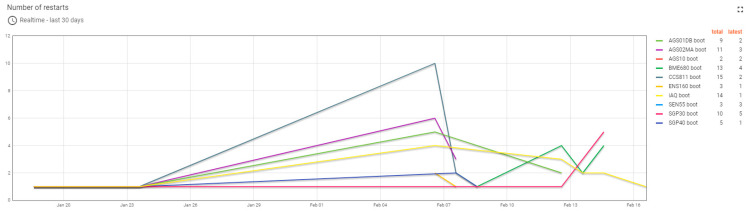
The graph of the evolution of the number of restarts for the ten test systems in the last 30 days.

**Figure 9:**
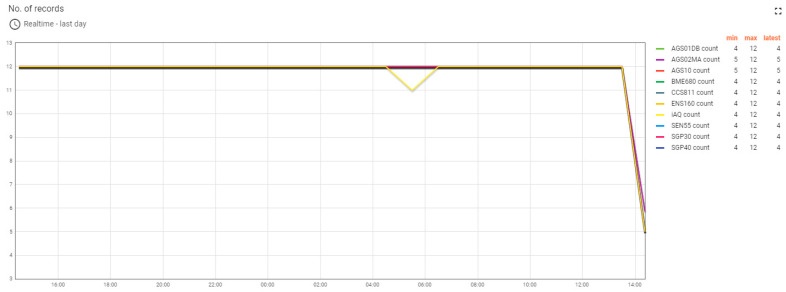
The graph of the number of registrations per hour for the ten test devices in the last 24 h.

Dashboards built on the ThingsBoard platform allowed for monitoring of the progress of the experiment both locally and remotely. The ThingsBoard server could also be accessed from outside the institutional network via the Internet. Monitoring the progress of the experiment allowed for viewing of the recorded values (Figure 6) and also the status of the monitored devices. The monitoring of the test devices involved the verification of the MQTT connection between the test devices and the ThingsBoard server (Figure 7), the stability of the operation of the test systems by counting the number of restarts for each system (Figure 8), and the good functioning of the data sending through measuring the number of records per unit of time (Figure 9).

### 3.3. Tests

**Figure 10:**
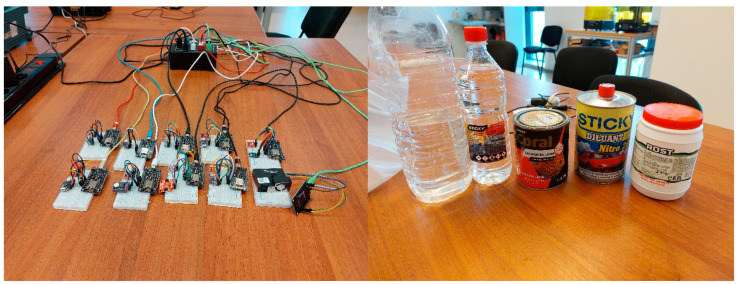
Image of the test system (**left**,**right**) containers with polluting agents (from right to left paper glue, D509, wood varnish, D209, ethanol) used for testing.

The test devices (Figure 10) were placed grouped in a didactic laboratory with automatic based ventilation controlled at the building level (Building Management Systems) in an open space (except for the tests presented in Section 4.3). The laboratory where the testing took place has an area of 50 square meters. During the testing period, the laboratory was used as usual by the research team of which the authors of the article are part. The research team that works in the laboratory where the testing was carried out consists of six people.

The testing had three phases. The first phase took place over a period of 9 months (June 2023–February 2024) and assumed the understanding of how each sensor works, the initial burn-in for the sensors, the improvement of the test environment—the communication network, the test programs, implementation of the user interface for the supervision of the tests. At the end of the first test phase, two phases involving data collection and analysis were carried out (end of February 2024).

The second phase took place over a period of 24 h and followed the analysis of the behavior of the ten sensors in a common work environment. During the 24 h, the activity in the test laboratory took place as usual. Data were collected from the TVOC sensors (and from the eight adjacent humidity and temperature sensors) at an interval of 5 min. The data were saved within the ThingsBoard platform. The result was 288 recordings with 10 sections, one from each sensor. Each section contains the following parameters: temperature, humidity, TVOC concentration measured in parts per billion (ppb) or as an index (SEN55 and SGP40), eCO2 concentration expressed in parts per million (ppm) (BME680, CCS811, ENS160, iAQ-Core and SGP30). The obtained results are presented in Section 4.1 and Section 4.2.

The third phase took place in an interval of 8 h in which 6 common polluting agents from the domestic environment were used (Figure 10). The sensors were exposed to the six polluting agents in a small closed box (0.1 m^3^). Each exposure lasted 30 min. Between exposures, the sensors were placed in fresh air for at least 30 min. The reading of the sensors was performed at an interval of 1 min. A total of 480 recordings resulted, each recording containing the same 10 sections as in the previous phase. The obtained results are presented in Section 4.3.

Prior to the last two test phases, the TVOC sensors worked continuously for at least 7 days. This way, both the warm-up phase and the automatic calibration (establishment of the measurement baseline) were ensured for all sensors.

The complete numerical results of the last two test phases have been published on the Zenodo platform [64].

The testing carried out and presented in this paper did NOT aim to compare the performances of the ten sensors. It was NOT aimed to check the accuracy, sensitivity, or precision of the sensors. The tests carried out did NOT comply with any metrological methodology or any other test standard. The sole purpose of the tests performed was to determine the ease of use of the sensors. It was considered that the way the sensors behave in common situations is a good indicator of the ease of integration in new monitoring devices.

## 4. Results and Discussion

### 4.1. Statistical Analysis of Recorded Data

The analysis was carried out on the data from the second test phase in which the operation of the ten sensors was observed under normal conditions. The analyzed parameters were temperature and humidity (Table 3 and Table 4), TVOC (Table 5), and eCO_2_ (Table 6). The abbreviation AVG refers to the arithmetic mean of the series of values (the sum of all values divided by the number of values), MED to the median value of the series of values (the value in the middle of the range of values), Mode to the most frequently occurring value, STD to the standard deviation (average distance between the values and the arithmetic mean), MAX at the maximum value in the series of values, MIN at the minimum value in the series of values, and the variation is the difference between the maximum and the minimum.

In the case of the analysis performed for the temperature and humidity parameters, a variation of no more than 2 °C (temperature) and no more than 10% (humidity) can be observed between the sensors, regardless of whether we are talking about the HTU21D sensors or the sensors integrated in BME680 and SEN55. The range of variation, the arithmetic averages, and the median values are similar for all ten sensors. The recorded values are normal and comfortable from the point of view of the thermal index. The average temperature recorded during the test was 22.88 °C and humidity 31.24%.

In the case of the TVOC parameter, the sensors tested under identical conditions offered an extremely varied statistical behavior, not to say extremely confusing. The minimum recorded value varied between 0 ppb (AGS01DB) and 491 ppb (BME680), the maximum value between 9 ppb (AGS02MA) and 55,695 ppb (BME680), the average value between 7 ppb (AGS02MA) and 16,436 ppb (BME680), and the median value between 0 ppb (AGS01DB) and 3552 ppb (BME680). This first test snapshot gives us an image of the rather different way in which the ten tested sensors behave even in normal environmental conditions. This different behavior justifies the testing carried out in this paper and the attempt to understand how this problem can be approached in a simple way in order to design an IAQ monitoring system.

The eCO_2_ parameter variation in the data set recorded from the ten sensors had a slightly more grouped behavior; the parameter variation was less scattered. The variation differences between the TVOC and eCO_2_ parameters can be explained by the fact that the TVOC parameter was measured and the eCO_2_ parameter was calculated based on the TVOC parameter starting from the premise that the only source of TVOC and eCO_2_ in a room is human breathing, and the ratio between the two parameters in the exhaled air is known [9]. The way in which this concept is implemented by each individual manufacturer is not known, but considering the observations made following the TVOC/eCO_2_ correlation analysis (Tables 10 and 11), it is clear that the calculation formula is not a linear function. For this reason, it can be assumed that the calculation formula of the eCO_2_ parameter alters the variability of this parameter. The minimum values recorded vary between 400 ppm (SGP30) and 595 ppm (BME680), the maximum values between 444 ppm (SGP30) and 2578 ppm (BME680), the average values between 400 ppm (SGP30) and 1524 ppm (BME680), and the median values between 400 ppm (SGP30) and 1515 ppm (iAQ-Core). Even if the eCO_2_ parameter is a calculated parameter that is not measured, it can be observed that there is no direct correlation between the TVOC parameter and eCO_2_. The highest TVOC value does not generate an equivalent maximum eCO_2_ value, just like the lowest TVOC value does not generate a minimum eCO_2_ value.

These differences observed in the statistical analysis carried out are justified by several factors such as the MOX technology used in each individual sensor, the different sensitivity of each sensor, the self-calibration mode, and the period during which the self-calibration is performed. These details specific to each sensor are part of the problem of designing an IAQ monitoring device.

### 4.2. Correlation Analysis between Recorded Time Series

Another aspect followed in the second phase of the testing was the correlation between the values measured by the ten sensors. As shown in the previous section, the ten sensors provide quite different instantaneous values for the TVOC concentration/index; for this reason, we went further and wanted to check how the measured values evolve over time. To analyze the correlation between the measured values, the Pearson correlation was used (Equation (1), where x and y are the two-time series for which the correlation is checked, with the $\bar{x}$ and $\bar{y}$ being the average values).

(1) $$r=\frac{\sum \left(x-\bar{x})(y-\bar{y}\right)}{\sqrt{\sum {\left(x-\bar{x}\right)}^{2}\sum {\left(y-\bar{y}\right)}^{2}}}$$

The calculation of the Pearson correlation factor was carried out in the presented tables (Table 7, Table 8, Table 9, Table 10, Table 11 and Table 12), extracted from the supplementary data set [64], using the PEARSON function from the Microsoft Excel 365 version 2402 environment. The correlation factor calculation function received two input series of values corresponding to the sensor whose name is on the line and column of the table. The output of the correlation function takes values between −1 and 1 and must be interpreted as follows: values close to 1, good correlation between the series of values; values around 0, non-correlation between the series of values; values close to −1, anti-correlation. In Table 7, the value series was used for the temperature parameter; in Table 8, the time series was used for the humidity parameter, in Table 9 for the TVOC parameter, in Table 10 for the eCO_2_ parameter and only for the five sensors that provide this parameter. All these tables have lines in the mirror of the columns, and for this reason, they have perfect correlation (=1) on the main diagonal: having the same sensor on the line and on the column verifies the correlation of a series with itself. Table 11 is not symmetrical, because it verifies the correlation between series of values of the eCO_2_ parameter (names at line level) and TVOC (names at column level). In Table 12 shows the TVOC parameter following exposure to a high concentration of ethanol (also a symmetrical table).

As can be seen from Table 7 and Table 8, the correlation between the temperature and humidity values of the ten test devices is almost perfect (>0.9). It is true that eight of the ten sensors are identical (HTU21D sensors), but the correlation is maintained even for the sensors integrated in the BME680 and SEN55 models.

The correlation between the series of values for TVOC shows differences in behavior between the sensors. While some sensors have a similar evolution to most of the others (AGS02MA, AGS10, CCS811, SEN55, SGP30 have an average correlation of over 0.8), there is also the opposite case (the AGS01DB sensor with an average correlation of just over 0.3). These correlations may indicate an easier integration into an IAQ monitoring device that has a similar behavior to other monitoring devices. It can also be noted that, as a group of manufacturing companies, the SEN55, SGP30, and SGP40 sensors behave very similarly, even if, in the case of the SEN55 and SGP40 sensors, we are not talking about the TVOC concentration but the index.

Only five out of ten sensors also provide values for the eCO_2_ concentration, and therefore, the correlation analysis, as well as the statistical analysis, was performed only for them. In the case of the series of values for eCO_2_, considering the previous statistical analysis that indicates a smaller range of variation, the correlation is not as good as in the case of TVOC. As a benchmark, it can be observed that the values provided by the SGP30 sensor are not correlated at all with the values of the other sensors, even if the correlation for the TVOC of this sensor is good. This indicates that in addition to the technological differences in the realization of the sensors, they also differ in the calculation algorithms implemented.

A final correlation analysis was performed between the TVOC and eCO_2_ concentration series for the five sensors that provide both parameters. The correlation between the own TVOC/eCO_2_ time series is good (>0.9, 1 for iAQ-Core) for four out of five sensors (less SGP30). This confirms that the eCO_2_ parameter has a calculated value but that the calculation is not based on a simple linear dependence formula. The correlation differences between the series of values also suggest a different implementation of the eCO_2_ calculation method between the five sensors.

### 4.3. Sensor Testing to Various Common Polluting Agents

**Figure 11:**
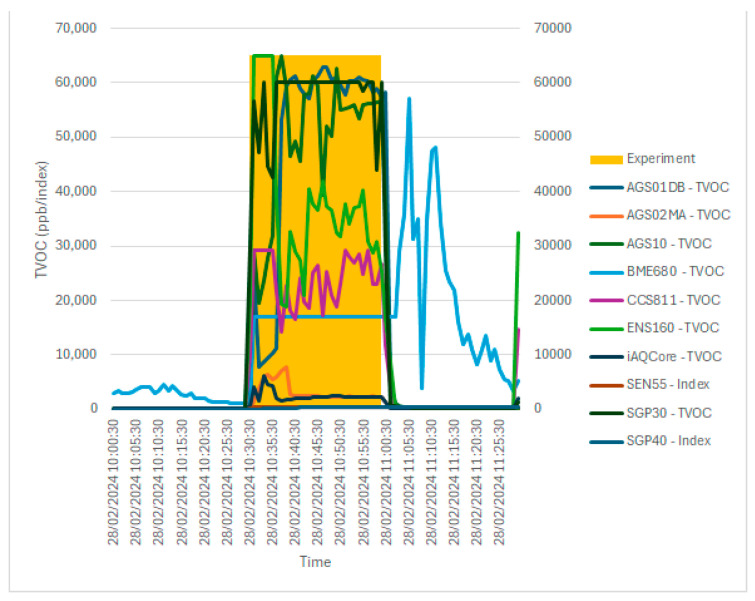
The evolution of the TVOC parameter as a result of exposure to a high concentration of ethanol in the air (the actual exposure period is highlighted in the graph with a different colored background).

In the third phase of testing, the sensors were exposed to six common polluting agents that can be found in a household (alcohol/ethanol, paint solvents, glue, cigarette smoke). In the current section, the response of the sensors to the ethanol agent will be presented, but the complete results can be consulted at [64]. The sensors were exposed to the polluting agent for 30 min, and the TVOC parameter was recorded and analyzed at 1 min intervals (the test software was modified). The exposure was preceded and followed by intervals of 30 min of exposing the sensors to fresh air. Figure 11 and Table 13 show the response of the sensors obtained after running the test.

All ten sensors had a strong response to exposure to the polluting agent, but it was observed that several sensors indicated a constant value during the test (BME680—16,690 ppb, SEN55—index 500, SGP30—60,000 ppb), and others they recorded varied but had high-level values. In the case of the SEN55 and SGP30 sensors, a higher saturation of the measured values is obvious. In the case of the BME680 sensor, there is no saturation of the measurement, and it is interesting to highlight the compartment after exposure to the polluting agent. During the ventilation after the test, during the period of exposure again to clean air, the BME680 sensor recorded very large oscillations of the measured value (higher than during the test, even measuring TVOC concentrations of 57.126 ppm). This behavior can be assumed to be due to an internal self-calibration process generated by the large variation in the applied stimuli. Even so, it is a behavior that can generate confusion for users of an IAQ monitoring system.

The correlation between the variation in the values measured by the three sensors was generally good (>0.7) with three exceptions. BME680 had a bad correlation with the other sensors; this can be explained by the oscillation of the measured values that occurred after the exposure to the polluting agent. SEN55 and SGP40 also had an uncorrelated variation, which can be explained by the limitation related to reporting through an index and not the actual measurement of a concentration.

The statistical analysis of the series of recorded values confirms some of the previous observations (related to the saturation of the measurement). It is noted that certain sensors have large variations in the measurement range (AGS01DB—62,850 ppb, AGS10—64,857 ppb, BME680—56,055 ppb, ENS160—65,000 ppb, SGP30—60,000 ppb), some of them reached the maximum measurement range with or without entering saturation (ENS160—without saturation, SGP30—with saturation). The range of variation was reached during the period of exposure to the polluting agent (not BME680). Some of the sensors stand out for their modest ranges of variation (AGS02MA—7761 ppb, iAQ-Core—6121 ppb), which can be explained by the small measurement range (iAQ-Core) and probably by a specific technological characteristic (AGS02MA). The two sensors that do not report the measured value in concentration but use an index have entered (SEN55, index 500) or almost entered (SGP40, index 492) saturation, which is normal because the index is thought to be the maximum equivalent of an approximate concentration of 5500 ppb. This problem is detailed in the following section.

Overall, the test in which the sensors were exposed to a high concentration of ethanol proves that the polluting agent was detected by all the sensors, but their response differed considerably due to the technological specificity of each sensor. MOX sensors are not suitable for measuring high gas concentrations [5]; in addition, the measurement range in which a good response is guaranteed is different for each individual sensor. Significant variations in the composition of the gas mixture evaluated by the sensors can also trigger the dynamic recalibration of the measurement algorithm (the best example is provided by the BME680 sensor), and for this reason, it is not recommended to use low-cost sensors in environments where VOC/TVOC concentrations are significant.

### 4.4. Concentration vs. Index

One of the current trends observed in certain air quality sensors is to provide the user with a unitless air quality index value instead of a concentration value. This is useful at first sight for the user who can easily evaluate and compare the air quality on an absolute scale. The tested sensors offer two types of indexes. BME680 and ENS160 provide a generic air quality (IAQ) index that suggests a global approach to air quality without making a unique reference to the TVOC parameter. For this reason, this parameter for the two sensors was ignored in the analysis performed in this paper, which focused on the ability of the sensors to measure TVOC (as a concentration or as an index). Unfortunately, the two sensors offer different scales for the calculated index. ENS160 follows the AQI-UBA format (derived from the German Federal Environmental Agency guide) with integer values between 1 and 5 ([45] page 11) and five associated quality levels. BME680 offers an index between 0 and 500 with seven quality levels ([43] page 9).

The SEN55 and SGP40 sensors do not provide a value for the concentration of TVOC in the air but only an index for TVOC and refer to this parameter as the VOC Index between 1 and 500 ([49], page 9). In the case of both sensors, the VOC Index is calculated externally (outside the sensor) based on the raw signals provided by the sensors. Since we are talking about the same manufacturer and the same generation of sensors, it can be assumed that the two sensors are based on the same technology, which is also confirmed by the correlation of the variations in the recorded parameters presented in the previous sections. Unfortunately, an equation cannot be made between the VOC Index offered by the two sensors and the IAQ offered by BME680 or ENS160, and a direct comparison cannot be made between the index offered by ENS160 and the two sensors. The manufacturer Sensirion offers a way to calculate the VOC concentration based on the VOC Index [65], but the goal is to increase the market for sensors by aligning them with the building standards, not to make their use easier.

The fact that there is no clear equivalence between various index values offered by various manufacturers makes these values misleading for the user and complicates the implementation of an easy-to-understand monitoring system. Equivalence, correlation, and usefulness of index values for measuring air quality is a separate research topic and is beyond the scope of this paper.

### 4.5. Trends in the Development of MOX-Based Sensors for TVOC/VOC Measurement

One of the evolution trends of MOX-type VOC/TVOC sensors was already presented in the previous section. The attempt to eliminate the calibrated value for VOC/TVOC measurement in favor of a specific or generic index is visible. This trend is present at least for certain manufacturers and has different implementations depending on the manufacturer. Some manufacturers are trying to eliminate the concentration value in favor of a VOC index, while others are trying to offer an index type alternative together with the concentration value for VOC/TVOC. The utility and the gain in ease of use are difficult to estimate without a broader analysis than the one carried out in this paper.

**Figure 12:**
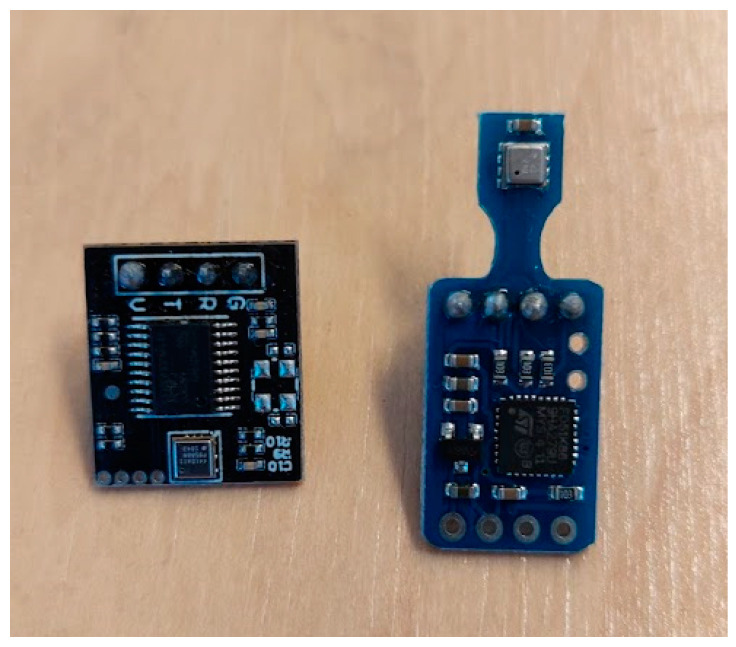
Serial modules with programmable electronic circuit included for ZMOD4410 (**left**) and BME680 (**right**) sensors.

Another trend observed in the tested sensors is the removal of the VOC/TVOC measurement algorithm from the sensor’s basic functionalities. The sensors (BME680, SEN55, SGP40) directly provide only the raw resistivity of the internal MOX sensor without a transformation into a concentration or index. The transformation takes place in software outside the sensor based on an algorithm provided by the manufacturer. Examples of similar approaches can be found in the Renesas ZMOD4410 sensor [66] and the Bosch BME688 sensor [67]; these sensors are not part of the tests presented in this paper. If the algorithm for the VOC index for the SEN55 and SGP40 sensors is available as source code on GitHub [68] under the BSD3 license, in the case of the BME68x and ZMOD4410 sensors, things are a little more restrictive. The algorithm for calculating TVOC concentration and IAQ for the BME68x sensor (BSEC Software, versions 1.x and 2.x) is only offered in binary form for certain architectures and under specific licensing clauses [69]. The use of an external library offers a lower price for the sensors and a greater functional flexibility without the need for firmware updates for the sensors, but it puts more pressure on the developers of the monitoring devices. There may be restrictions regarding the development hardware platform and the minimum required performances. Licensing the software components offered by the manufacturer may bring additional costs as well as the related technical support. For these reasons, an adjacent minor trend can be observed: sensor modules that put together the sensor and a programmable device that contains the necessary library, thus eliminating complications for the developer of the monitoring device. Figure 12 shows two such serial modules for the BME680 and ZMOD4410 sensors. Unfortunately, this solution introduces adjacent problems to be avoided: another software component in the system that must be maintained, an uncertain way of licensing the proprietary algorithms through an intermediary, and the loss of functional flexibility offered by the manufacturer’s software component.

A general trend, which goes beyond the context of VOC/TVOC sensors, is the integration of several sensors in a single chip or in a single measurement unit. This is the case of the BME680 sensor which is capable of measuring temperature, humidity, atmospheric pressure, and gaseous compounds and of the SEN55 sensor which is capable of measuring temperature, humidity, VOC index, NO_x_ index, and concentrations of PM_1.0_, PM_2.5_, PM_4.0_, PM_10_ (the future SEN6x family will also add CO_2_). Temperature and humidity are two important parameters in measuring air quality, especially for sensors that use these values for the compensation related to the MOX sensor. The integration of multiple sensors in a single measurement unit reduces the complexity and cost of the monitoring system, allowing a simpler and easier implementation.

One last trend is worth bringing to the attention of monitoring system developers, the integration of artificial intelligence algorithms in applications associated with VOC/TVOC sensors. This is the case of the BSEC2 library (BSEC version 2) for the BME68x sensors and the library provided by the manufacturer for the ZMOD4410 sensor. The purpose of integrating artificial intelligence algorithms into measurement algorithms is to increase measurement accuracy and even selective measurement for various gaseous compounds. Unfortunately, these objectives bring an associated computational cost that complicates and increases the cost of designing and implementing monitoring systems.

## 5. Conclusions

The MOX technology for measuring the concentration of various gases in the air represents a low-cost solution for the implementation of various monitoring devices from gas detectors to air quality devices. Digital MOX-based VOC/TVOC sensors, like those presented and tested in this paper, represent a low-cost solution for air quality monitoring systems, allowing a simple and fast hardware design.

The analysis and testing of the ten MOX-based VOC/TVOC digital sensors carried out in this paper also highlighted certain difficulties that must be carefully managed by the designers and users of the monitoring systems. Unlike other environmental parameters, such as temperature and humidity, measuring the level of a concentration in the air of a gaseous compound through MOX technology requires the understanding and compliance of certain specific elements such as the following:

- initial burn-in time for sensors and start-up time (warm-up).
- establishing a reference level (baseline) by exposure to a good quality environment (clean air) when initializing the sensor.
- the lifetime of the sensor.
- measurement compensation depending on other environmental parameters (for example, temperature and humidity).
- respecting the measurement range and recognizing the phenomenon of upper saturation, unlike the temperature parameter where the upper measurement limit is not normally reached; in the case of indoor gas accumulation, there is a risk of higher concentrations than the sensor can measure.

A challenge for designers and users of air quality monitoring devices based on TVOC MOX sensors is represented by the substantial differences between the values measured by different sensors. These differences can be generated by an incorrect use that does not take into account the specific elements listed above and due to different sensor manufacturing technologies. Additional research is needed to explore and validate a solution for equating the measured results. It is possible that the introduction of a numerical index will lead to a sensor-independent evaluation tool in the future, but this requires additional rigorous and careful evaluation which is beyond the scope of the current paper.

Despite the specific elements, the tested sensors showed that there is the possibility of easy integration of the current MOX-based digital sensors that measure VOC/TVOC for the implementation of effective indoor air quality monitoring systems. The correlation of the measured values proved that even if there are technological differences, all the sensors behave similarly and can generate a monitoring system with common elements for the user.

The identified development trends are promising and represent a hope of significant improvement for future generations of sensors that will offer increased integration and superior accuracy. All tested sensors proved good reliability and stable behavior during nine months of intensive testing. All this makes us confident that the use of these sensors can be the starting point for widely adoptable monitoring devices that impose user-friendly technologies for IAQ management.

## Figures and Tables

**Table 1:** List of evaluated sensors, target physical parameters, initial release dates, and manufacturer-recommended applications.

	Sensor Name	Manufacturing Company	Target Physical Parameters	Initial Official Release/ Last Revision Date for Datasheet	Applications/Typical Applications (Extracted Directly from the Datasheet of Each Sensor)
1	AGS01DB	Aosong Electronic ^1^	TVOC	May 2018/July 2018 [40]	Air purifiers, home appliances, fresh air system.
2	AGS02MA	Aosong Electronic ^1^	TVOC	July 2022 [41]	Air purifiers, home appliances, fresh air system.
3	AGS10	Aosong Electronic ^1^	TVOC	July 2022 [42]	Air purifiers, home appliances, fresh air system.
4	BME680	Bosch ^2^	Temp RH Pressure TVOV	June 2022 [43]	Indoor air quality, home automation and control, Internet of things, Weather forecast, GPS enhancement, Indoor navigation, Outdoor navigation, leisure and sports applications, Vertical velocity indication
5	CCS811	ScioSense (AMS AG) ^3^	TVOC eCO2	December 2016 [44]	This device can be mainly used for indoor air quality monitoring in: Smart phones, Wearables, Home and Building automation, Accessories.
6	ENS160	ScioSense ^3^	TVOC eCO2	November 2019/ March 2023 [45]	Building Automation/smart home/HVAC (Indoor air quality detection, Demand-controlled ventilation, Smart thermostats), Home appliances (Cooker hoods, Air cleaners/purifiers), IoT devices
7	IAQ Core	ScioSense (AMS AG) ^3^	TVOC eCO2	v1-00/April 2015 [46]	Smart Home, Internet of Things, HVAC, Thermostats
8	SEN55	Sensirion ^4^	Temp RH VOC NOx PM	January 2022/ March 2022 [47]	HVAC and Air Quality Applications
9	SGP30	Sensirion ^4^	TVOC eCO2	May 2020 [48]	Indoor air quality applications. Easy integration into air purifier, demand-controlled ventilation, and IoT applications.
10	SGP40	Sensirion ^4^	VOC	July 2020/February 2022 [49]	Indoor air quality applications. Easy integration into air purifier s or demand-controlled ventilation systems.

^1^ Guangzhou Aosong Electronic Co., Ltd. No. 17, Yunjun Road, Huangpu District, 510530 Guangzhou, China. ^2^ Robert Bosch GmbH, Robert-Bosch-Platz 1, 70839 Gerlingen-Schillerhöhe, GERMANY. ^3^ Sciosense B.V., High Tech Campus 10, 5656 AE Eindhoven, The Netherlands. ^4^ Sensirion AG, Laubisruetistrasse 50, 8712 Stäfa, Switzerland.

**Table 2:** Summary of sensor characteristics and applications.

	Sensor Name	Dynamic Range	Resolution (ppm)	Accuracy (Compound Tested)	Response Time (s)	Cost ($)	Applications Examples/References
1	AGS01DB [37]	0–100 ppm	0.1 [37]	20% (ethanol)	≥2	<3$	[52]
2	AGS02MA [38]	0–100 ppm	Unspecified	25% (ethanol)	≥2	<5$	[53]
3	AGS10 [39]	0–100 ppm	Unspecified	25% (ethanol)	≥2	<3$	No scientific research reported
4	BME680 [40]	1–500 AQI	1 AQI	15% S2S (ethanol, bVOC)	<1	<10$	[54,55,56,57,58,59,60]
5	CCS811 [41]	0–1187 ppb (TVOC), 400–8192 ppm (eCO2)	Unspecified 16-bit ADC	Not specified	0.25	<5$	[6,7,9]
6	ENS160 [42]	0–65,000 ppb (TVOC) 400–65,000 ppm (eCO2) 1–5 AQI	1 ppb 1 ppm 1 AQI	12% (hydrogen)	1	<10$	[54]
7	IAQ Core [43]	125–600 ppb (TVOC) 450–2000 ppm (eCO2)	Unspecified	Not specified	<10	<20$	[55]
8	SEN55 [44]	1–500 AQI	Unspecified	15% S2S (ethanol)	1	<30$	[56]
9	SGP30 [45]	0–60,000 ppb (TVOC) 400–60,000 ppm (eCO2)	1, 6, 32 ppb 1, 3.9, 31 ppm	15% (ethanol) 10% (hydrogen)	1	<10$	[28,36,57,58]
10	SGP40 [46]	1–500 AQI	Unspecified	15% (ethanol)	1	<7$	[54]

**Table 3:** Analysis of the temperature data set.

Sensor	Temperature AVG	Temperature MED	Temperature Mode	Temperature STD	Temperature MAX	Temperature MIN	Temperature Variation
AGS01DB (HTU21D)	21.90	21.68	21.30	0.71	23.56	21.13	2.43
AGS02MA (HTU21D)	22.63	22.45	22.52	0.70	24.57	21.85	2.72
AGS10 (HTU21D)	22.86	22.62	22.62	0.70	24.79	22.11	2.68
BME680	23.42	23.20	22.76	0.72	25.12	22.64	2.47
CCS811 (HTU21D)	23.36	23.12	23.10	0.62	25.22	22.57	2.66
ENS160 (HTU21D)	23.47	23.23	23.21	0.67	25.22	22.75	2.46
IAQ (HTU21D)	23.51	23.28	23.04	0.64	25.37	22.62	2.76
SEN55	21.90	21.83	21.94	0.64	23.48	21.17	2.31
SGP30 (HTU21D)	23.30	22.96	22.88	0.66	25.44	22.40	3.03
SGP40 (HTU21D)	22.47	22.28	21.87	0.68	24.30	21.72	2.58

**Table 4:** Analysis of the humidity data set.

Sensor	Humidity AVG	Humidity MED	Humidity Mode	Humidity STD	Humidity MAX	Humidity MIN	Humidity Variation
AGS01DB (HTU21D)	35.31	35.61	38.17	2.57	39.33	30.28	9.05
AGS02MA (HTU21D)	33.39	33.58	35.82	2.49	37.34	28.16	9.17
AGS10 (HTU21D)	32.86	33.25	35.73	2.56	36.95	27.58	9.37
BME680	30.59	30.81	32.84	2.04	33.91	26.63	7.28
CCS811 (HTU21D)	31.34	31.73	33.50	2.22	35.26	26.48	8.78
ENS160 (HTU21D)	23.56	23.99	21.60	2.05	26.96	19.34	7.62
IAQ (HTU21D)	24.06	24.42	25.93	1.99	27.48	19.76	7.72
SEN55	35.41	35.35	37.64	1.92	38.56	31.48	7.08
SGP30 (HTU21D)	31.39	32.09	32.01	2.30	35.59	26.03	9.56
SGP40 (HTU21D)	34.51	34.78	35.52	2.25	38.14	29.88	8.27

**Table 5:** Analysis of the TVOC data set.

Name	TVOC AVG	TVOC MED	TVOC Mode	TVOC STD	TVOC MAX	TVOC MIN	TVOC Variation
AGS01DB	115.39	0.00	0.00	208.68	710.00	0.00	710.00
AGS02MA	7.52	7.00	9.00	1.19	9.00	6.00	3.00
AGS10	65.21	61.75	93.00	24.50	95.00	34.30	60.70
BME680	16,436.84	3552.15	569.40	19,393.43	55,695.29	491.90	55,203.39
CCS811	118.43	63.20	5.30	110.80	300.90	0.00	300.90
ENS160	193.03	206.20	239.30	48.41	283.20	88.70	194.50
IAQ	385.83	418.70	527.50	172.26	608.40	125.70	482.70
SEN55 (index)	103.15	63.10	203.00	69.51	203.70	30.00	173.70
SGP30	45.99	35.50	19.40	30.00	88.40	0.40	88.00
SGP40 (index)	62.53	22.55	12.00	60.93	163.00	10.00	153.00

**Table 6:** Analysis of the eCO_2_ data set.

Name	eCO2 AVG	eCO2 MED	eCO2 Mode	eCO2 STD	eCO2 MAX	eCO2 MIN	eCO2 Variation
BME680	1524.47	1360.69	2565.33	756.54	2578.00	595.80	1982.20
CCS811	1004.75	818.75	1609.80	499.17	1662.40	401.70	1260.70
ENS160	680.06	700.55	736.30	64.85	785.50	529.60	255.90
IAQ	1395.63	1515.00	889.20	625.60	2204.20	451.50	1752.70
SGP30	400.36	400.00	400.00	3.50	444.30	400.00	44.30

**Table 7:** Pearson correlation table for the ten series of temperature values. The green color indicates a strong correlation.

TEMP	AGS01DB	AGS02MA	AGS10	BME680	CCS811	ENS160	iAQ-Core	SEN55	SGP30	SGP40
**AGS01DB**	1	0.991699	0.987383	0.998808	0.977116	0.992416	0.987442	0.989598	0.96967	0.993288
**AGS02MA**	0.991699	1	0.995103	0.994005	0.985002	0.993659	0.988735	0.986922	0.976414	0.999169
**AGS10**	0.987383	0.995103	1	0.989818	0.989275	0.995974	0.985159	0.975833	0.980115	0.996597
**BME680**	0.998808	0.994005	0.989818	1	0.978486	0.993353	0.986633	0.989896	0.970128	0.995062
**CCS811**	0.977116	0.985002	0.989275	0.978486	1	0.991816	0.98805	0.966495	0.98973	0.985479
**ENS160**	0.992416	0.993659	0.995974	0.993353	0.991816	1	0.9895	0.981098	0.982868	0.9955
**iAQ-Core**	0.987442	0.988735	0.985159	0.986633	0.98805	0.9895	1	0.979017	0.989641	0.989039
**SEN55**	0.989598	0.986922	0.975833	0.989896	0.966495	0.981098	0.979017	1	0.95098	0.987
**SGP30**	0.96967	0.976414	0.980115	0.970128	0.98973	0.982868	0.989641	0.95098	1	0.977026
**SGP40**	0.993288	0.999169	0.996597	0.995062	0.985479	0.9955	0.989039	0.987	0.977026	1

**Table 8:** Pearson correlation table for the ten series of humidity values. The green color indicates a strong correlation.

HUMID	AGS01DB	AGS02MA	AGS10	BME680	CCS811	ENS160	iAQ-Core	SEN55	SGP30	SGP40
**AGS01DB**	1	0.997315	0.996344	0.999184	0.993623	0.997465	0.996162	0.99658	0.99057	0.997765
**AGS02MA**	0.997315	1	0.998442	0.997851	0.995949	0.997568	0.996224	0.99638	0.991997	0.999659
**AGS10**	0.996344	0.998442	1	0.996851	0.997926	0.998949	0.996054	0.994188	0.993994	0.998979
**BME680**	0.999184	0.997851	0.996851	1	0.993964	0.997632	0.996084	0.996963	0.990946	0.99834
**CCS811**	0.993623	0.995949	0.997926	0.993964	1	0.997851	0.996282	0.990138	0.996695	0.996277
**ENS160**	0.997465	0.997568	0.998949	0.997632	0.997851	1	0.997114	0.993968	0.995201	0.998386
**iAQ-Core**	0.996162	0.996224	0.996054	0.996084	0.996282	0.997114	1	0.992865	0.996841	0.996676
**SEN55**	0.99658	0.99638	0.994188	0.996963	0.990138	0.993968	0.992865	1	0.984124	0.996819
**SGP30**	0.99057	0.991997	0.993994	0.990946	0.996695	0.995201	0.996841	0.984124	1	0.992529
**SGP40**	0.997765	0.999659	0.998979	0.99834	0.996277	0.998386	0.996676	0.996819	0.992529	1

**Table 9:** Pearson correlation table for the ten series of TVOC concentration values/indexes. From green to orange, from strong correlation to anticorrelation.

TVOC	AGS01DB	AGS02MA	AGS10	BME680	CCS811	ENS160	iAQ-Core	SEN55	SGP30	SGP40
**AGS01DB**	1	0.254817	0.390833	0.532391	0.175483	−0.00092	0.526315	0.338325	0.321577	0.012081
**AGS02MA**	0.254817	1	0.929027	0.85401	0.970279	0.686379	0.840928	0.941338	0.941555	0.923158
**AGS10**	0.390833	0.929027	1	0.850293	0.91572	0.592695	0.957222	0.917506	0.889363	0.856209
**BME680**	0.532391	0.85401	0.850293	1	0.847709	0.449376	0.832594	0.940817	0.894306	0.766756
**CCS811**	0.175483	0.970279	0.91572	0.847709	1	0.641925	0.828203	0.968223	0.958003	0.973589
**ENS160**	−0.00092	0.686379	0.592695	0.449376	0.641925	1	0.40332	0.55786	0.640979	0.577431
**iAQ-Core**	0.526315	0.840928	0.957222	0.832594	0.828203	0.40332	1	0.873417	0.836406	0.75142
**SEN55**	0.338325	0.941338	0.917506	0.940817	0.968223	0.55786	0.873417	1	0.97381	0.919811
**SGP30**	0.321577	0.941555	0.889363	0.894306	0.958003	0.640979	0.836406	0.97381	1	0.89687
**SGP40**	0.012081	0.923158	0.856209	0.766756	0.973589	0.577431	0.75142	0.919811	0.89687	1

**Table 10:** Pearson correlation table for the five series of eCO_2_ values (only the sensors that provide the eCO_2_ parameter). From green to orange, from strong correlation to anticorrelation.

eCO2	AGS01DB	AGS02MA	AGS10	BME680	CCS811	ENS160	iAQ-Core	SEN55	SGP30	SGP40
**AGS01DB**										
**AGS02MA**										
**AGS10**										
**BME680**				1	0.962165	0.529631	0.928501		−0.04209	
**CCS811**				0.962165	1	0.630692	0.865876		−0.03732	
**ENS160**				0.529631	0.630692	1	0.390728		0.13529	
**iAQ-Core**				0.928501	0.865876	0.390728	1		−0.00421	
**SEN55**										
**SGP30**				−0.04209	−0.03732	0.13529	−0.00421		1	
**SGP40**										

**Table 11:** Pearson correlation table between TVOC and eCO_2_ value series (only the sensors that provide the eCO_2_ parameter). From green to orange, from strong correlation to anticorrelation.

eCO2/TVOC	AGS01DB	AGS02MA	AGS10	BME680	CCS811	ENS160	iAQ-Core	SEN55	SGP30	SGP40
**AGS01DB**										
**AGS02MA**										
**AGS10**										
**BME680**				0.929904	0.927457	0.536933	0.9285		0.942818	
**CCS811**				0.884055	0.986478	0.639984	0.865882		0.964221	
**ENS160**				0.450441	0.630037	0.998313	0.390723		0.629599	
**iAQ-Core**				0.832609	0.828192	0.403321	1		0.836394	
**SEN55**										
**SGP30**				−0.07303	−0.0507	0.151411	−0.0042		0.016523	
**SGP40**										

**Table 12:** Correlation table for the evolution of the TVOC parameter as a result of exposure to ethanol (the correlation matrix is symmetrical with respect to the first diagonal). From green to orange, from strong correlation to anticorrelation.

Pearson	AGS01DB	AGS02MA	AGS10	BME680	CCS811	ENS160	iAQ-Core	SEN55	SGP30	SGP40
AGS01DB	1	0.623073	0.9517841	0.216116	0.8212362	0.6610571	0.66004092	0.485318	0.910934	0.320382
AGS02MA		1	0.7785496	0.205455	0.806561	0.7884756	0.806492017	0.453133	0.816595	0.077032
AGS10			1	0.231299	0.894269	0.7597803	0.757723441	0.515935	0.962565	0.277967
BME680				1	0.2129567	0.1916036	0.185304216	0.688824	0.225274	0.637923
CCS811					1	0.9426917	0.91251034	0.522662	0.951564	0.20224
ENS160						1	0.941782659	0.485681	0.86903	0.111028
iAQ-Core							1	0.455686	0.85121	0.105208
SEN55								1	0.520766	0.889802
SGP30									1	0.212892
SGP40										1

**Table 13:** Statistical data of the values recorded during exposure to ethanol.

Name	TVOC AVG	TVOC MED	TVOC Mode	TVOC STD	TVOC MAX	TVOC MIN	TVOC Variation
AGS01DB	16,765.08	50.00	0.00	26,208.38	62,850.00	0.00	62,850.00
AGS02MA	1081.57	25.75	7.00	1802.50	7768.20	7.00	7761.20
AGS10	16,696.98	98.50	48.00	24,533.03	64,897.00	39.50	64,857.50
BME680	13,144.33	13,144.33	13,144.33	13,144.33	57,126.00	1071.00	56,055.00
CCS811	8253.42	75.25	50.50	11,482.54	29,206.00	25.00	29,181.00
ENS160	13,290.31	246.75	65,000.00	19,447.72	65,000.00	0.00	65,000.00
IAQ	988.51	988.51	127.50	1232.34	6246.50	125.00	6121.50
SEN55	337.69	482.25	500.00	215.16	500.00	33.00	467.00
SGP30	19,331.27	153.50	60,000.00	27,127.79	60,000.00	0.00	60,000.00
SGP40	275.92	374.25	492.00	212.93	492.00	10.00	482.00

## Data Availability

The data presented in this study are openly available in Zenodo at https://zenodo.org/doi/10.5281/zenodo.10782316, reference number [64].
